# Prevention of Histological Changes after Colonic Diversion in Rats: An Experimental Study

**DOI:** 10.21699/jns.v6i2.511

**Published:** 2017-04-15

**Authors:** Pathak M, Srinivas M, Shariff A

**Affiliations:** 1 Department of Pediatric Surgery, All India Institute of Medical Sciences, Jodhpur, 342001, India; 2 Department of Pediatric Surgery, All India Institute of Medical Sciences, New Delhi, 110029, India; 3 Department of Anatomy, All India Institute of Medical Sciences, New Delhi, 110029, India

**Keywords:** Histology, Colonic diversion, Glutamine, Psyllium, Short Chain Fatty Acids, Maharishi Amrit Kalash, Enema

## Abstract

**AIM:**

To determine the beneficial effects of Glutamine, Psyllium, Short Chain Fatty Acids (SCFA), and Maharishi Amrit Kalash (MAK), in preventing the histological changes after diversion colostomy.

**MATERIAL AND METHODS:**

After ethical clearance, male wistar rats (n=40) underwent diversion colostomy. Rats were divided into five groups of 8 rats each. Each group was given, after diversion colostomy, per rectally, one of the five agents being tested as an enema (3 ml/kg/day). Group I: Normal saline. Group II: Glutamine Group III: Psyllium, Group IV: MAK. Group V: Short chain fatty acid. The rats were euthanised 45 days after performing diversion colostomy. Morphometrical analysis of defunctionalised colon was done. Statistical analysis was done using SSPS statistical analysis software.

**RESULTS:**

On comparison with Group I epithelial cell height and mucosal thickness was significantly higher in Group II. Muscularis externae thickness was significantly higher in Group III on comparison with Group I. Group V had least inflammatory changes.

**CONCLUSIONS:**

Atrophic and inflammatory changes in the diverted colon can be prevented by per rectal administration of Glutamine, Psyllium and Short chain fatty acids.

## INTRODUCTION

Diversion of the colon may be required during the management of various diseases like anorectal malformation, Hirschsprung’s disease etc. If the fecal stream is diverted as in diversion colostomy, the colon may undergo various degenerative changes [1]. Multiple agents have been described to be essential for mucosal health [2-5].

 Whether these agents will be able to prevent the changes in diverted colon is still being debated. This animal study was designed to test the role of glutamine, short chain fatty acids, psyllium, and Maharishi Amrit Kalash (MAK) in diverted colon.

## MATERIALS AND METHODS

This experiment was performed after obtaining permission from Institutional Animal Ethics Committee (IAEC). Forty 60 days old male rats were included in the study. Diversion colostomy was performed under general anesthesia using ketamine hydrochloride (50mg/kg) intra-peritoneally. The ketamine was given in step-up doses till adequate anesthesia was achieved. The abdomen was prepared and painted with povidone-iodine. All surgical procedures were performed, under antibiotic cover (ceftriaxone- 100mg/kg) and strict asepsis. A two-centimeter-long mid-line incision was made and the caecum delivered through this incision. The colon was divided 5 cm distal to the caecum in all cases. A proximal stoma and distal mucus fistula were created, 3 cm apart in the midline, using single layer, interrupted, colo-musculo-cutaneous sutures (5-0 prolene). The wide separation of the stoma prevented faecal spillage into the distal segment. The abdomen was closed in 2 layers after instilling 4 ml of sterile normal saline intra-peritoneally to make up for the intra-operative fluid losses. The wound was dusted with Neosporin powder and cages were cleaned twice for 7 days after surgery. All the rats were kept under daily supervision till they were euthanized.

Rats were divided into five groups of 8 rats each as described below. Each group was given every day, as 3 ml/kg enema, one of the five agents being tested. Colonic enemas started from the next day of surgery.

Group I: Control: Normal saline.

Group II: Glutamine (Claris, Ahmedabad, India) : 1000mg/kg/day.

Group III: Psyllium (Fibril, Micronised natural fiber from Psyllium husk, Lupin, Mumbai, India): 3000 mg / kg/day.

Group IV: MAK (Maharishi Ayurveda, Delhi, India) : 5 mg/kg/day 

Group V: Short chain fatty acids (Sigma Aldrich labs, India).

### Tissue Collection

All the rats were re-operated 45 days after performing diversion colostomy and the above mentioned enemas. Abdomen was opened and the defunctionalised colon was collected from 1 cm distal to the site of mucus fistula up to the anus. The tissue collection, processing, staining and morphometry has been published in detail from our laboratory [6]. Briefly, the intestine was kept undisturbed for five minutes in 0.1 M phosphate till no visible contractions were seen. In the defunct colonic segment, the lumen had no contents. The lumen of proximal colon was cleaned gently with 0.1 M phosphate buffer. The tissues were immediately processed for further studies. This ensured that the sharpest images of the stained cells were obtained. The pieces of colonic tissue from the region of interest were processed, in order to facilitate the visualization of the micro-anatomical details of the gut wall.

### Tissue fixation and processing

The colonic tissues were fixed, dehydrated, cleared and embedded in paraffin wax for cutting serial sections of the tissue. Briefly stated below are the steps of processing:

1. Fixation in 4% Para formaldehyde for 7 days.
2. Washed in tap water for 6 hours.
3. Dehydration in ascending grades of alcohol (50%-absolute alcohol).
4. Clearing in cedar wood oil for 5-7 days
5. Two dips in xylene for 5 minutes each prior to embedding in paraffin wax (xylene-wax, and 2 changes in wax only) at 600 C0.

Serial sections of 5 micrometer thickness were cut from these blocks on a rotary microtome. The sections were mounted on clean glass slides coated with egg albumin (egg albumin and thymol). All the tissue processing was done with extreme care to minimize damage to the tissues.

### Staining

Haematoxylin and eosin was used for staining the tissues.

### Morphometry

Stained sections were examined under the microscope. Below mentioned parameters were looked for in each group:

1. Epithelial cell height; 2. Mucosal thickness; 3. Muscularis mucosae thickness; 4. Muscularis externa thickness; 5. Grade of inflammation – Inflammation was classified subjectively into mild, moderate, and severe grades.

### Statistical analysis 

Data was entered on excel worksheet. Analysis was done using SSPS statistical software. Descriptive statistics were noted down. Comparison between two groups was done by Mann-Whitney test. P value< 0.05 was considered significant.

## RESULTS

**Figure 1:**
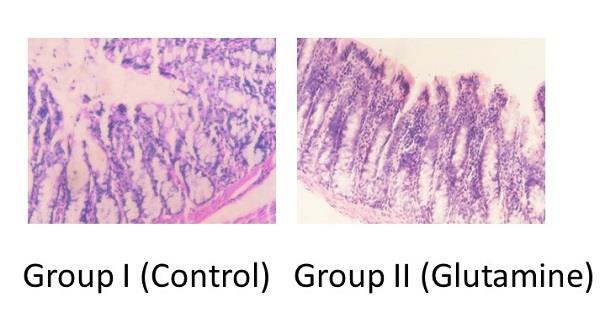
Photomicrographs comparing mucosal cell height and thickness. Glutamine group is showing increased mucosal cell height and thickness. (Haematoxylin & Eosin stain, Magnification 20X).

There was no statistical difference in epithelial cell height and mucosal thickness of Group I in comparison with Groups III, IV, and V (Table 1, Figure 1) though the parameters were much higher in Group II.

**Figure 2:**
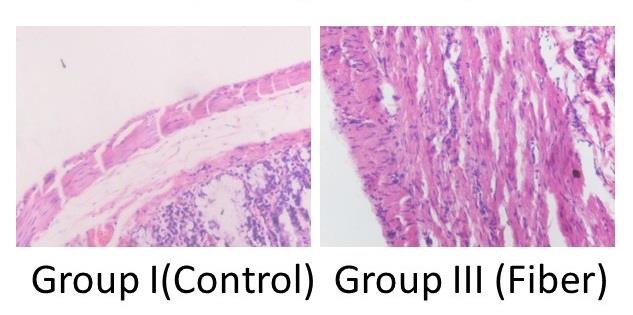
Photomicrographs comparing thickness of muscularis externae. Fibre group is showing increased thickness of muscularis externa. (Haematoxylin & Eosin stain, Magnification 20X).

Maximum muscularis externae thickness was in Group III. Muscularis externae thickness was significantly higher in Group III on comparison with Group I (Figure 2). There was no significant difference in muscularis externae thickness of Group II, IV, and V in comparison with Group I (Table 2).

**Figure 3:**
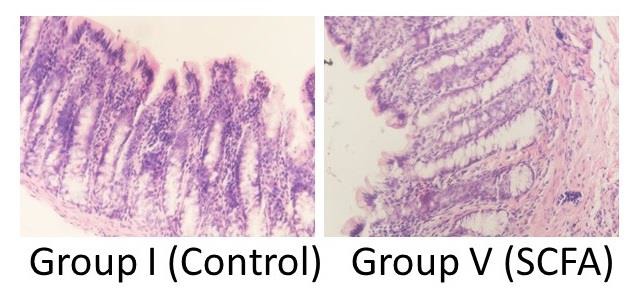
Photomicrographs comparing the presence of inflammatory cells in the mucosa and submucosa. SCFA group is showing decreased inflammation. Haematoxylin & Eosin stain, Magnification 20X).

Seven rats (88%) in Group I had severe and one rat (12%) had moderate grade of inflammation. Five rats (62%) had severe and three rats (38%) had moderate grade of inflammation in Groups II, III, and IV. No rat in any group had mild grade of inflammation except Group V. All rats in Group V had mild grade of inflammation (Figure 3) only.

## DISCUSSION

It is well established that diversion colostomy leads to atrophic changes in the distal colon. We designed this study to verify the ability of the various agents in preventing these changes after diversion colostomy in rats. Our study has demonstrated the effectiveness of glutamine in preventing the mucosal atrophy. This study has revealed the beneficial effects psyllium on muscularis externae of diverted colon. In addition, the present study has confirmed the inflammatory changes in diverted colon are most effectively be prevented by Short Chain Fatty Acids (SCFA).

L-Glutamine is a fuel source for the epithelial cells of colon [3,4,7]. It is an essential precursor for nucleic acid bio-synthesis and is therefore essential for proliferation of cells with rapid turnover such as the intestinal mucosa. 

The present study has found that epithelial cell height, and mucosal thickness was significantly more in Group II when compared with Group I. Only 62% rats in Group II had severe grade of inflammation as compared to 88% in Group I rats. Thus glutamine preserved the epithelial cell height and prevented the mucosal atrophy in the diverted colon. It also reduced the severity of the grade of inflammation. But glutamine did not have any beneficial effect on muscularis externae. 

 Paulo *et al*. studied the effects of oral supplement of L-glutamine on the diverted colonic wall [8]. They found that colostomy caused a significant reduction of the crypts length and the supplement of L-glutamine was able to avoid this change. Blachier *et al*. also have studied the effect of L-glutamine on muscularis externae and found that there was no significant difference between glutamine and control group [9]. The present study endorses these findings. 

Dietary fibers are required for proper colonocytes functioning. SCFA are produced by fermentation of fibers. Thus fibers act as a substrate to provide the colonocytes a fuel source in the form of SCFA [1,3]. The present study has found that there was no significant difference in epithelial cell height and mucosal thickness of Group III that received Psyllium. Muscularis externae thickness was highest in Group III. Only 62% rats had severe grade of inflammation in diverted colon in comparison to 88% in control group. Thus psyllium prevented the muscular atrophy in diverted colon. It also reduced the number of rats having severe grade of inflammation. However, psyllium did not have any significant effect on epithelial cell height or mucosal thickness.

Oliveira-Neto *et al*. studied the effect of intraluminal irrigation with fibers on diverted colon [10]. They concluded that irrigation with fibers improves inflammation at the defunctionalised colon. Our results also show the beneficial effect of psyllium on inflammatory changes in diverted colon, but in addition we have found the beneficial effect of psyllium on thickness of muscularis externae. This finding has surgical implications – for the success of the surgical re-anastomosis and post-operative colonic motility.

SCFA are the main oxidative energy source for the colonic mucosa. All SCFA (acetate, propionate, and butyrate) are suitable substrates but butyrate is considered to be the preferred energy source for colonocytes. SCFA have trophic effects on the intestinal mucosa and stimulate colonic sodium and water absorption. Role of SCFA on maintenance of colonic health has been extensively studied. It has been ascribed as the fuel for colonocytes. According to Mortenson and Clausen [2], it has trophic effect on colonic mucosa. Post-operative instillation of SCFA has been suggested to reduce the risk of leakage due to an increased microcirculation flow to the resection edges. On these principles, the present animal experiment tested the effects of SCFA on diverted colon. There was no significant difference in epithelial cell height, mucosal thickness, and muscularis externae thickness of Group V. SCFA were found to be most effective only in preventing the inflammatory changes in diverted colon. All rats in Group V had mild inflammation only, while rats in all other groups had either moderate or severe inflammation. Kissmeyer *et al*. studied the trophic effect of SCFA on diverted colon [1, 11]. They found significant increases among SCFA-treated rats in the weight of the mucosa, the sub mucosa, and the muscularis propria. Measurements of breaking strength and hydroxyproline content showed no differences between treatment groups. Friedel *et al*. also studied the effects of SCFA on diverted colon [12]. Adult male rats were maintained on total parenteral nutrition (TPN) and, in addition, received either 150 mmol/L of saline or 150 mmol/L of SCFA mixture (60:25:15, acetate: propionate: butyrate) into the proximal colon. SCFA infusion into the colon had no significant effect on absorptive function. However, significantly greater mucosal height and mucosal DNA were observed. Thus our study differs from Kissmeyer *et al*. [11] and Friedel *et al*. [12] in not finding beneficial effect on epithelial cell height, mucosal thickness, and muscularis externae.

Harig *et al*. studied four patients with diversion colitis [13]. Instillation of a solution containing SCFA twice daily resulted in the disappearance of the symptoms and inflammatory changes observed at endoscopy over a period of 4 to 6 weeks. Interruption of treatment for 2 weeks resulted in definite worsening. In striking contrast to the effects of the SCFA solution, the effects of the enemas containing the physiological saline, given twice daily for 2 to 4 weeks, was only a small decrease in the endoscopic score. The histological changes resolved more slowly and less completely than gross appearance of the inflamed mucosa. Contrary to it, Guillemot *et al*. did not find any histological improvement in diversion colitis by SCFA irrigation [14]. Kiely *et al*. studied the response to treatment with SCFA in diverted colon of five children [15]. They found that SCFA provided effective relief of symptoms. Thus our results showing reduction in inflammation in distal colon are similar to the Harig *et al*. [13] and Kiely *et al*. [15], but differ from published work of Guillemot *et al*. [14].

MAK has immune-modulant and anti-oxidant properties similar to glutamine [5]. We tried to test this basic information and MAK was given for defunctionalised colon. No significant beneficial effect on epithelial cell height and mucosal thickness was found. There was no significant effect on muscularis externae also. MAK was found to be effective in reducing` the inflammation in the diverted colon. Sixty two percent of MAK treated rats had severe inflammation in comparison to 88% in control group. MAK has been studied for its anticancer properties, liver regeneration, and rejuvenation of central nervous system, but no study has tested its role in diverted colon. Probably MAK reduces the inflammation by its antioxidant and immuno-modulant properties. Glutamine having similar properties was found to be beneficial in preventing mucosal atrophy. It seems that the beneficial effect of glutamine on mucosa is not due to its antioxidant property or immuno-modulant effect. That may be the reason for MAK not being found effective in preventing the mucosal atrophy. 

The agents utilized in the present study do not have any side effects. These agents can safely be used to maintain the overall well-being of mucosa and muscle in diverted colon. These agents can be used in diverted patients to revert the atrophic and inflammatory changes in diverted colon. This may reduce the chances of anastomotic breakdown and complications of colostomy closure. 

Short chain fatty acids may be tried to reduce the incidence of enterocolitis. Children who undergo successful surgical interventions for Hirschsprung disease continue to have enterocolitis post operatively. They may be offered SCFA enemas to reduce this life threating complication.

## CONCLUSIONS

Glutamine prevents the mucosal atrophy in diverted colon. Psyllium prevents the muscular atrophy in diverted distal colon. Short chain fatty acids are most effective in decreasing the severity of inflammation in diverted colon. Hence to have the best protection after diversion of colon a combination of the glutamine, SCFA and psyllium may be considered.

In clinical practice, it would be interesting to study whether these changes after colostomy are reversible and whether these agents reduce the incidence of complications following colostomy closure.

## Footnotes

**Source of Support:** None

**Conflict of Interest:** None

## Figures and Tables

**Table 1:** Epithelial cell height (microns) and mucosal thickness (microns) in various groups

	Epithelial cell height Mean +/- SD (‘p’ value)	Mucosal thickness Mean+/-SD (‘p’ value)
Group I	11.86 +/- 2.57	211.60 +/- 55.99
Group II	17.38 +/- 6.21 (<0.05)	275.31+/-43.86 (<0.05)
Group III	11.93 +/- 2.35 (NS)	208.90 +/- 19.03 (NS)
Group IV	13.36 +/- 2.55 (NS)	207.66 +/- 41.04 (NS)
Group V	12.51+/- 2.78 (NS)	208.32 +/- 20.64 (NS)

**Table 2:** Muscularis externae thickness (microns) in various groups.

	Mean +/- SD	‘p’ value
Group Ic	82.95 +/- 32.60	
Group II	120.62 +/- 37.89	NS
Group III	172.65 +/- 35.38	<0.05
Group IV	86.97 +/- 15.89	NS
Group V	85.91 +/- 30.38	NS
